# Genomic Regions Related to White/Black Tail Feather Color in Dwarf Chickens Identified Using a Genome-Wide Association Study

**DOI:** 10.3389/fgene.2021.566047

**Published:** 2021-04-30

**Authors:** Changsheng Nie, Liang Qu, Xinghua Li, Zhihua Jiang, Kehua Wang, Haiying Li, Huie Wang, Changqing Qu, Lujiang Qu, Zhonghua Ning

**Affiliations:** ^1^State Key Laboratory of Animal Nutrition, Department of Animal Genetics and Breeding, National Engineering Laboratory for Animal Breeding, College of Animal Science and Technology, China Agricultural University, Beijing, China; ^2^Jiangsu Institute of Poultry Science, Chinese Academy of Agricultural Sciences, Yangzhou, China; ^3^Department of Animal Sciences, Washington State University, Pullman, WA, United States; ^4^College of Animal Science, Xinjiang Agricultural University, Urumqi, China; ^5^College of Animal Science, Tarim University, Xinjiang, China; ^6^Key Laboratory of Tarim Animal Husbandry Science and Technology, Xinjiang Production and Construction Corps, Xinjiang, China; ^7^Engineering Technology Research Center of Anti-aging Chinese Herbal Medicine of Anhui Province, Fuyang Normal University, Fuyang, China

**Keywords:** dwarf chicken, tail feather color, inheritance pattern, genetics, genome-wide association study

## Abstract

Although the genetic foundation of chicken body feather color has been extensively explored, that of tail feather color remains poorly understood. In the present study, we used a synthetic chicken dwarf line (DW), derived from hybrids bred between a black tail chicken breed, Rhode Island Red (RIR), and a white tail breed, dwarf layer (DL), to investigate the genetic rules associated white/black tail color. Even though the body feathers are predominantly red, the DW line still comprises individuals with black or white tails after more than 10 generations of self-crossing and selection for the body feather color. We first performed four crosses using the DW chickens, including black-tailed males to females, reciprocal crosses between the black and white, and white males to females to elucidate the inheritance pattern of the white/black tail. We also performed a genome-wide association (GWA) analysis to determine the candidate genomic regions underlying the tail feather color using black tail chickens from the RIR and DW lines and white individuals from the DW line. In the crossing experiment, we found that (i) the white/black tail feather color is independent of body feather color; (ii) the phenotype is a simple autosomal trait; and (iii) the white is dominant to the black in the DW line. The GWA results showed that seven single-nucleotide polymorphisms (SNPs) on chromosome 24 were significantly correlated with tail feather color. The significant region (3.97–4.26 Mb) comprises nine known genes (*NECTIN1*, *THY1*, *gga-mir-1466*, *USP2*, *C1QTNF5*, *RNF26*, *MCAM*, *CBL*, and *CCDC153*) and five anonymous genes. This study revealed that the white/black tail feather trait is autosome-linked in DW chickens. Fourteen genes were found in the significant ~0.29 Mb genomic region, and some, especially *MCAM*, are suggested to play critical roles in the determination of white/black tail feather color. Our research is the first study on the genetics underlying tail feather color and could help further the understanding of feather pigmentation in chickens.

## Introduction

Tail feather color can be different from body feather color in birds. Compared with body feather color, the genetic basis of chicken tail feather color remains poorly defined. Tail feather color is a naturally and sexually selected trait in chickens, as well as in wild birds such as the rock sparrow (Griggio et al., 2011), barn swallow (Kose and Møller, 1999), and peacock (Weiss and Kirchner, 2010), and is combined with artificial selection in poultry, especially chickens. Black and white are predominant tail feather colors in chickens; however, some chicken breeds also display red, blue, yellow, purple, or multi-colored tail feathers.

Feather color is a genetically complex trait, the foundation of which has been extensively explored in birds (Delmore et al., 2016; Cooke et al., 2017), especially chickens. The dominant white, dun, and smoky colors are associated with the *PMEL17* polymorphism (Kerje et al., 2004). Mutations in *MLPH* causes the dilution of both black eumelanin and red/brown pheomelanin pigments (Vaez et al., 2008). Furthermore, more than one gene, such as *TYR* (Chang et al., 2006; Dorshorst et al., 2010) and *SLC45A2* (Gunnarsson et al., 2007), can be responsible for white feather color. The extended black plumage is associated with *MC1R* (Kerje et al., 2003; Dávila et al., 2015; Charoensook et al., 2017). The sex-linked barring feather pattern is controlled by the *CDKN2A/B* locus (Hellstrom et al., 2010).

**Figure 1:**
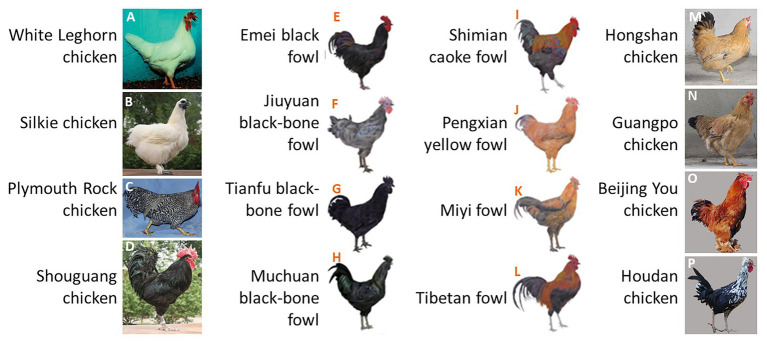
Chicken feather color. **(A–H)** Body feather color is consistent with tail feather color; **(I–P)** body feather color is different from tail feather color. Image Source: **(A)** Kerje et al. (2004), **(B,D)** Feng et al. (2014), **(C)** Hellstrom et al. (2010), **(E–L)** Li et al. (2019), **(M,N)** Wang et al. (2017), and **(O,P)** Zhang et al. (2016).

Relatively few studies have investigated the inheritance of chicken tail color as an isolated trait. Geneticists normally regard tail color as part of the body plumage color because the tail color is strongly intertwined with body feather color in some chicken breeds. White chickens always have white tails (Figures 1A,B), individuals with barred plumage always have barred tails (Figure 1C), and black cockerels also have black tails (Figures 1D–H). However, the segregation of tail feather and body feather colors is widely represented in some breeds (Figures 1I–P). Additionally, the daughters of male Rhode Island Red (RIR; with sex-linked recessive red plumage and a black tail) and female Rhode Island White (RIW; with sex-linked dominant white plumage) present red body feathers and white tail color, indicating that body feather color and tail color are controlled by different genes in these chickens. We also observed that dwarf line (DW) hybrids generated from more than 10 generations of self-crossings between RIR and a white-tailed dwarf layer (DL) line contain both white- and red-tailed individuals, even though the hybrids were selected for red body feather color. Therefore, in this study, we used this population to investigate the genetic basis of white/black tail phenotypes in chickens.

## Materials and Methods

### Animals

**Figure 2:**
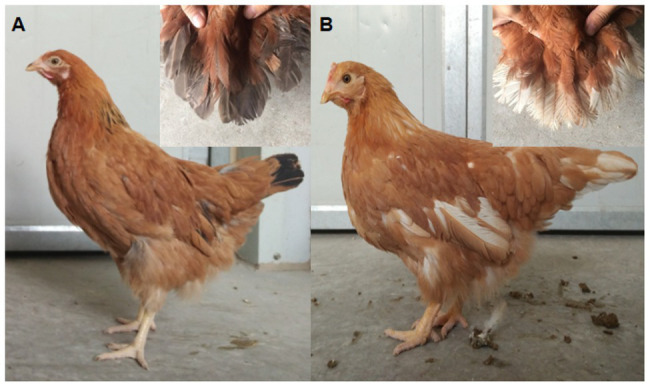
Dwarf hens displaying white or black tail feather color. **(A)** Black tail; **(B)** white tail.

The birds used in this study were derived from RIR and DL chicken populations (Beinongda commercial breeding farm and Jiangsu Institute of Poultry Science experiment farm). The RIR, a dual-purpose commercial breed, has red body plumage and black tail plumage, and the red feather color is determined by a Z chromosome-linked recessive allele. The DL chicken is a layer line with white plumage, which is defined by a Z-linked dominant allele. The DW chickens with white and black tail feathers were generated through more than 10 generations of self-crossings of RIR and DL. Because only the red body feather color was selected in each generation, independently of tail color, the DL population presents both black and white tail feathers, whereas most display red body plumage (Figure 2).

### Inheritance Pattern of Tail Feather Color

To explain the inheritance pattern of the tail colors, four crosses were performed using the black-tailed DW and white-tailed DW: black × black (cross 1), black males × white females (cross 2), white males × black females (cross 3), and white × white (cross 4). Two replicates of the four crosses (crosses 5–8) were used to confirm the results. Tail feather color was identified at 7 weeks of age when the tail feathers emerged.

### Mapping the Genomic Region Underlying White/Black Tail Feather Color by a GWAS

A total of 176 adult female chickens were selected, including 96 black-tailed RIRs and 80 DWs (38 black-tailed and 42 white-tailed) to perform the GWAS (Figure 2). Blood samples from both populations were collected from the wing vein and placed into centrifuge tubes containing anticoagulating agent.

Genomic DNA was extracted using phenol/chloroform (Green and Sambrook, 2017), and genotyping was performed using a 600 K Affymetrix Axiom Chicken Genotyping Array (Affymetrix, Inc. Santa Clara, CA, United States; Kranis et al., 2013). Affymetrix Power Tools v1.16.0 (APT) software was then used for quality control and genotype calling. Specifically, only samples with dish quality control >0.82 and call rate >97% were used in the subsequent analysis.

Single-nucleotide polymorphisms (SNPs) with a minor allele frequency <1% or a *p*-value of deviation from Hardy-Weinberg equilibrium (PHWE) <1 × 10^−6^ were removed. Ultimately, 175 individuals and 479,579 SNPs were retained for the association analysis. Classical multi-dimensional scaling analysis was used to detect the population structure using PLINK v1.09 software (Purcell et al., 2007).

### Statistical Analysis

To test the association of each SNP with tail feather color, a mixed model (Price et al., 2010) association analysis was used, including fixed effects (overall mean and covariates) and random effects (SNP effect, individual effect, and residual errors), according to the GEMMA (v0.94.1) manual (Zhou and Stephens, 2012). In the present study, 175 genotyped birds were obtained from two different populations; therefore, the first two principal components (accounting for 23.89 and 2.31% of the total variability) were used as a covariate to account for population structure in the analysis.

All the selected SNPs were subjected to linkage disequilibrium analysis, using the *--indep-pairwise 25 5 0.2* and *--blocks-max-kb 500* commands in PLINK, to generate a pruned subset of 48,848 SNPs and 77,137 haploblocks with linkage equilibrium. Bonferroni adjustment is a widely used method for multiple hypothesis testing. Given the correlation between the SNPs in linkage disequilibrium, the traditional Bonferroni adjustment appears to be overly conservative, with the key assumption that all the tests are independent (Johnson et al., 2010). Herein, the sum of independent blocks plus singleton markers was used to define the number of independent statistical tests (Nicodemus et al., 2005; Gu et al., 2011). With this approach, 125,985 independent tests were suggested to determine the *p*-value threshold. Consequently, the genome-wide significant and suggestive *p*-values were 3.97 × 10^−7^ (0.05/125,985) and 7.94 × 10^−6^ (1/125,985), respectively. To further location candidate region that affect trait, we performed linkage disequilibrium (LD) analysis with genome significantly SNPs in Haploview software (v4.2; Barrett et al., 2005).

In addition, the annotated genes were identified using the NCBI and Ensembl annotations of the *Gallus Ensemble* version 5.0 genome. A Manhattan plot of genome-wide *p*-values of the association analysis was created using R.^1^

## Results

### White Tail Feather Color in Dwarf Chickens Is an Autosome-Linked Dominant Character

**Figure 3:**
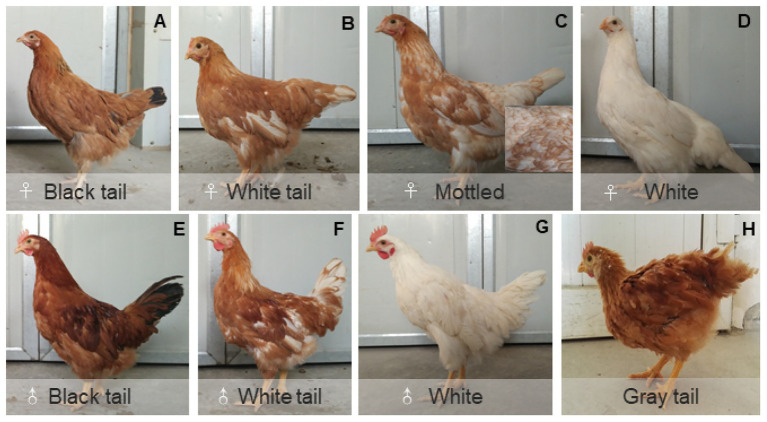
Main feather color subtypes in the offspring of Dwarf chickens. **(A–D)** The different feather color phenotypes of Dwarf hens; **(E–G)** the different feather color phenotypes of Dwarf cockerels. **(H)** Some offspring have gray tail feathers.

We first made four crosses between white-tailed and black-tailed chickens and the chickens all showed red body feather color. Because a few outliers were identified in the four crosses, we generated the other four crosses to confirm our results. Almost all the offspring presented red body plumage, with a few exceptions where the body feather color was white (Figure 3). Because body feather color might affect tail feather color, we only used the offspring with red body plumage to understand the inheritance pattern of white/black tail color. Table 1 presents the distribution of white or black tail progenies in the eight crosses.

**Table 1:** Progeny phenotypes of eight crosses in the Dwarf chicken population.

Cross	Parents		Black-tailed offspring		White-tailed offspring		Mottled offspring		Gray-tailed offspring
	Male	Female	Male	Female	Male	Female	Male	Female	
1	B^1^	B	66	58	1^3^	0	0	0	/
2	B	W^2^	26	36	49	39	0	7	/
3	W	B	26	27	14	27	0	6	/
4	W	W	6	8	53	62	0	20	/
5	B	B	146		1^3^		/		0
6	B	W	68		74		/		7
7	W	B	33		46		/		2
8	W	W	16		104		/		4

1 Black tail feather.

2 White tail feather.

3 Outlier, assuming that white tail feather color in the DW chicken is an autosome-linked dominant trait.

We found that the white/black tail feather color is a Mendelian trait, and the white is dominant to the black (Table 1). The same results were obtained with both replicates. Because the red body feather and tail feather colors were segregated, we concluded that the genes controlling white/black tail color were different from those controlling red body feather color; additionally, there was no epistatic effect between them.

### Candidate Genes on Chromosome 24 Identified by the GWAS

**Figure 4:**
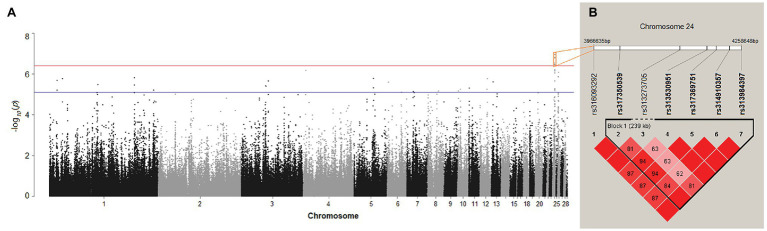
**(A)** Manhattan plot showing the association of all single-nucleotide polymorphisms (SNPs) with the tail feather color (white/black) trait of Dwarf and Rhode Island Red chickens. SNPs were plotted on the *x*-axis according to their position on each chromosome against their association with these traits on the *y*-axis (shown as −log10 *p*-values). The red and blue lines indicate the genome-wide and suggestive significant association with *p*-values of 3.97 × 10^−7^ (0.05/125,985) and 7.94 × 10^−6^ (1/125,985), respectively. **(B)** Linkage disequilibrium (r2) plot association with white/black tail feather color.

After quality control, 175 female chickens were analyzed, 134 (76%) of which presented black tail feather color as the controls, and 41 (24%) presented white tail feather color as the cases. Based on the Manhattan plot for the white/black tail feather color, we observed seven significantly associated SNPs spanning from 3.97 to 4.26 Mb (~0.29 Mb) on chromosome 24 (*p* < 3.97 × 10^−7^) in the sexually mature hens (Figure 4A; Table 2). The linkage disequilibrium plot (Figure 4B) showed the detected SNP markers were strongly linked in a haplotype block. Moreover, 14 candidate genes were found to be related to tail feather color, including nine annotated genes and five anonymous genes, namely: *NECTIN1*, *THY1*, *gga-mir-1466*, *USP2*, *C1QTNF5*, *RNF26*, *MCAM*, *CBL*, *CCDC153*, *ENSGALG00000046117*, *ENSGALG00000006746*, *ENSGALG00000037367*, *ENSGALG00000032979*, and *ENSGALG00000039907*. Additionally, 36 autosomal SNPs were suggestively related (7.94 × 10^−6^) to the white/black tail feather in chicken (Figure 4A; Supplementary Table S1).

**Table 2:** Single-nucleotide polymorphisms (SNPs) significantly associated with the tail feather color in the genome-wide association study (GWAS).

SNP	GGA^1^	Position^2^ (bp)	Minor/major	*p*-value	MAF^3^		Candidate genes (location)	Full name	Functions
					DW	RIR			
rs314910357	24	4,235,437	T/C	1.12e-07	0.14	0.00	*CBL* (intron)	Cbl proto-oncogene	B cell receptor endocytosis and ligand-induced signaling (Jacob et al., 2008).
rs313984397	24	4,258,648	C/T	1.54e-07	0.16	0.00	*CCDC153* (intron)	Coiled-coil domain containing 153	NA
rs317369751	24	4,209,809	T/C	1.64e-07	0.11	0.00	*MCAM* (intron)	Melanoma cell adhesion molecule	Coordination of morphogenesis (Gao et al., 2017) and endothelial adhesion (Guezguez et al., 2007).
							*RNF26* (downstream 7.14 kb)	Ring finger protein 26	Lysosomal positioning and movement (Cabukusta and Neefjes, 2018).
rs313530951	24	4,190,968	A/G	1.65e-07	0.11	0.00	*ENSGALG00000032979* (intron)	NA	NA
							*C1QTNF5* (upstream 2.25 kb)	C1q and TNF related 5	Disease-related (Schwartze et al., 2017; Stanton et al., 2017; Dinculescu et al., 2018).
							*USP2* (downstream 3.82 kb)	Ubiquitin specific peptidase 2	Cell growth or death and disease-related (Zhu and Gao, 2017).
							*ENSGALG00000039907* (upstream 8.51 kb)	NA	
							*THY1* (downstream 17.10 kb)	Thy-1 cell surface antigen	Myofibroblast apoptosis (Liu et al., 2017).
							*gga-mir*-1466 (downstream 17.03 kb)	gga-mir-1466	NA
rs316093292	24	3,966,635	T/C	3.00e-07	0.06	0.00	*NECTIN1* (upstream 65.13 kb)	Nectin cell adhesion molecule 1	Disease-related (Takahashi et al., 2018), hair follicle morphogenesis (Hayashi et al., 2016).
rs313273705	24	4,137,245	G/A	3.46e-07	0.08	0.00	*ENSGALG00000006746* (downstream 47.62 kb)	NA	NA
							*ENSGALG00000037367* (downstream 37.50 kb)	NA	NA
rs317350539	24	4,018,982	G/A	3.62e-07	0.13	0.00	*ENSGALG00000046117* (upstream 13.32 kb)	NA	NA

DW, Dwarf line; RIR, Rhode Island Red line.

1 Chicken chromosome.

2 Position of SNPs according to the Gallus_gallus-5.0 primary reference genome assembly.

3 Minor allele frequency.

## Discussion

Tail feather color (white/black) in DW chickens is a qualitative trait, and we assumed that it was controlled by a single gene. The results of our crossing experiments supported our assumption, and revealed that the white tail feather color in DWs is an autosome-linked dominant trait. However, the crosses produced some offspring with white body plumage and gray tail feathers (Table 1), which has two possible explanations. First, the body and tail feather colors are not controlled by the same gene in DW chickens. Second, an intermediate feather color existed in the population at an early developmental period, and most of the heterozygous individuals were classified as having gray tail feathers; however, a few progeny might have been erroneously classified as black- or white-tailed.

We also aimed to locate positional candidate genes associated with tail feather color using a 600 K SNP panel for genotyping DW and RIR chickens. We identified 14 candidate genes in the most significant region on chromosome 24, which corresponded to nine known and five anonymous genes.

One candidate gene, *melanoma cell adhesion molecule* (*MCAM*), which encodes an endothelial adhesion receptor or an independent receptor for fibroblast growth factor 4, was identified as playing an essential role in lymphocyte endothelium interactions and morphogenesis (Guezguez et al., 2007; Gao et al., 2017). Melanocytes are derived from melanoblasts that originate from neural crest cells in early chicken embryos (Yu et al., 2004), and fibroblasts can influence melanogenesis (Muriel et al., 2010; Kim et al., 2016). Furthermore, Mangahas et al. (2004) reported that human *MCAM* is involved in primary melanocyte development *via* endothelin upregulation. The endothelin 3 locus has been reported to be responsible for hyperpigmentation in chickens (Dorshorst et al., 2011). Moreover, the tumor suppressor locus cyclin-dependent kinase inhibitor 2A/B can affect pigmentation phenotypes in the chicken (Hellstrom et al., 2010). Together, these observations indicate that *MCAM* may play an important role in the determination of tail feather color, a possibility that warrants future validation in the chicken.

Besides the promising candidate gene *MCAM*, other candidate genes were also identified in this ~0.29 Mb region, and have various functions (Table 2). For example, *C1QTNF5*, *USP2*, and *NECTIN1* have been reported as being disease-associated (Hayashi et al., 2016; Stanton et al., 2017; Zhu and Gao, 2017; Takahashi et al., 2018). *CBL* can promote B cell receptor endocytosis and attenuate ligand-induced signaling (Jacob et al., 2008), while *RNF26* was found to be associated with lysosomal positioning and movement (Cabukusta and Neefjes, 2018). *THY1* is correlated with myofibroblast apoptosis (Liu et al., 2017).

Currently, the mechanism underlying tail feather color remains almost unknown. However, the GWAS results of this study may contribute to determining the relationship between these candidate genes and tail feather color. Further research is necessary to determine the genetic basis underlying tail feather color in chickens.

## Conclusion

Our study showed that the white/black tail feather trait is autosome-linked in DW chickens. In addition, the GWAS revealed seven significant SNPs spanning a ~0.29 Mb region on GGA24 associated with the tail feather color in DW chickens, corresponding to 14 genes. Notably, among these 14 genes, *MCAM* may play a critical role in the formation of white/black tail feather color. Overall, the candidate genes detected herein can help elucidate the genomic architecture underlying white/black tail feather color and provide novel insights into the mechanisms regulating feather color development in DW chickens and other breeds.

## Data Availability Statement

The data of this study have been uploaded on the NCBI database with the BioProject ID: GSE130568.

## Ethics Statement

The animal study was reviewed and approved by Ministry of Agriculture of China (Beijing, China), Animal Welfare Committee of China Agricultural University (Beijing, China). Written informed consent was obtained from the owners for the participation of their animals in this study.

## Author Contributions

LuQ, ZN, and KW conceived and designed the experiments. CN, CQ, and LiQ performed the experiments. CN and XL analyzed the data. CN wrote the manuscript. ZJ, HL, HW, and LuQ revised the manuscript. All authors read and approved the final version of the manuscript.

### Conflict of Interest

The authors declare that the research was conducted in the absence of any commercial or financial relationships that could be construed as a potential conflict of interest.

## Footnotes

^1^https://www.r-project.org/
