# Supplementary material for: Genomic Regions Related to White/Black Tail Feather Color in Dwarf Chickens Identified Using a Genome-Wide Association Study
Source: Front Genet. 2021 Apr 30;12:566047. doi: 10.3389/fgene.2021.566047 (PMC8120320; doi:10.3389/fgene.2021.566047)
Supplement: Supplementary file 1 [file Table_1.docx]

Table S1. Suggestive SNPs associated with the tail feather color phenotype

| SNP | | GGA^1^ | Physical Position^2^  (bp) | Allele | *P*-value |
| --- | --- | --- | --- | --- | --- |
| Probe Set ID | dbSNP rs ID |  |  |  |  |
| AX-76311919 | rs317663314 | 24 | 4,018,982 | A/G | 6.33E-07 |
| AX-76667743 | rs313806888 | 4 | 4,289,161 | A/C | 6.67E-07 |
| AX-76311463 | rs315522229 | 24 | 4,371,819 | A/G | 7.13E-07 |
| AX-76333002 | rs312679298 | 26 | 4,175,948 | A/G | 8.35E-07 |
| AX-76311753 | rs314937125 | 24 | 1,668,889 | T/C | 9.08E-07 |
| AX-76332260 | rs317176493 | 26 | 4,246,692 | A/G | 1.37E-06 |
| AX-80880581 | rs312877286 | 26 | 1,495,267 | C/G | 1.39E-06 |
| AX-76332482 | rs316605050 | 26 | 1,500,839 | T/G | 1.40E-06 |
| AX-80958142 | rs13960346 | 1 | 1,542,948 | A/T | 1.54E-06 |
| AX-76824336 | rs14531747 | 5 | 152,949,564 | A/G | 1.64E-06 |
| AX-75410139 | rs314513225 | 1 | 34,955,093 | T/C | 1.69E-06 |
| AX-75688213 | rs315991435 | 12 | 23,329,242 | T/C | 1.69E-06 |
| AX-75275087 | rs313154777 | 1 | 13,475,157 | A/G | 2.06E-06 |
| AX-76311967 | rs317644118 | 24 | 13,834,742 | T/C | 2.21E-06 |
| AX-76488993 | rs315647987 | 3 | 4,300,196 | T/C | 2.23E-06 |
| AX-80853600 | rs313834208 | 13 | 47,840,740 | C/G | 2.49E-06 |
| AX-80814393 | rs315398197 | 6 | 3,920,008 | C/G | 2.50E-06 |
| AX-75540580 | rs13648121 | 1 | 2,588,422 | A/C | 3.36E-06 |
| AX-75299286 | rs13960371 | 1 | 86,939,041 | T/C | 3.45E-06 |
| AX-76478981 | rs317796348 | 3 | 152,971,279 | T/C | 3.82E-06 |
| AX-76480794 | rs317489735 | 3 | 43,164,431 | A/G | 3.98E-06 |
| AX-76824394 | rs316617982 | 5 | 44,022,980 | A/G | 4.79E-06 |
| AX-75183987 | --- | 11 | 34,975,774 | T/C | 5.04E-06 |
| AX-76311670 | rs315977666 | 24 | 1,126,098 | A/G | 5.41E-06 |
| AX-76311733 | rs317200609 | 24 | 4,226,450 | T/C | 5.66E-06 |
| AX-75613514 | rs315259952 | 10 | 4,240,654 | T/C | 5.69E-06 |
| AX-81003249 | rs312894794 | 12 | 5,501,321 | A/T | 5.69E-06 |
| AX-75377988 | rs315059118 | 1 | 12,835,736 | A/G | 6.25E-06 |
| AX-80809295 | rs312882587 | 1 | 187,428,093 | A/T | 6.28E-06 |
| AX-76311727 | rs314273331 | 24 | 13,842,820 | A/G | 6.46E-06 |
| AX-77080039 | rs317359629 | 8 | 4,238,994 | A/G | 6.92E-06 |
| AX-75581669 | rs312271671 | 10 | 20,160,672 | A/G | 6.96E-06 |
| AX-75581839 | rs314890580 | 10 | 1,700,511 | T/C | 6.96E-06 |
| AX-75581884 | rs312899571 | 10 | 1,705,115 | T/C | 6.96E-06 |
| AX-75581890 | rs316919502 | 10 | 1,706,251 | A/G | 6.96E-06 |
| AX-76975341 | rs312943591 | 7 | 1,706,414 | A/G | 7.45E-06 |

^1^Chicken chromosome.

^2^Position of SNPs according to the Gallus_gallus-5.0 primary reference genome assembly.
